# Supplementary material for: Framework nucleic acids as programmable carrier for transdermal drug delivery
Source: Nat Commun. 2019 Mar 8;10:1147. doi: 10.1038/s41467-019-09029-9 (PMC6408537; doi:10.1038/s41467-019-09029-9)
Supplement: Supplementary file 1 — Supplementary Information [file 41467_2019_9029_MOESM1_ESM.pdf]

Supporting Information

Framework Nucleic Acids as Programmable Carrier for Transdermal Drug Delivery

Wiraja et al

Framework Nucleic Acids as Programmable Carrier for Transdermal Drug Delivery

Christian Wiraja[a,#], Ying Zhu[b,#], Daniel Lio[a], David C. Yeo[a], Mo Xie[b], Weina Fang[c], Qian Li[c], Mengjia Zheng[a], Maurice Van Steensel[d], Lihua Wang[b,e], Chunhai Fan[c,*], Chenjie Xu[a,f,g *]

[a] School of Chemical and Biomedical Engineering, Nanyang Technological University, 62 Nanyang Drive, 637459, Singapore

[b] Division of Physical Biology and Bioimaging Center, Shanghai Synchrotron Radiation Facility, CAS Key Laboratory of Interfacial Physics and Technology, Shanghai Institute of Applied Physics, Chinese Academy of Sciences, Shanghai 201800, China

[c] School of Chemistry and Chemical Engineering, and Institute of Molecular Medicine, Renji Hospital, School of Medicine, Shanghai Jiao Tong University, Shanghai 200240, China

[d] Lee Kong Chian School of Medicine, Nanyang Technological University, Singapore, 639798, Singapore.

[e] Shanghai Key Laboratory of Green Chemistry and Chemical Processes, School of Chemistry and Molecular Engineering, East China Normal University, 500 Dongchuan Road, Shanghai, 200241, China

[f] NTU-Northwestern Institute for Nanomedicine, Nanyang Technological University, 50 Nanyang Avenue, 639798, Singapore

[g] National Dental Centre of Singapore, 5 Second Hospital Ave, 168938, Singapore

[#]These authors contributed equally.

* Email correspondence to: fchh@sinap.ac.cn, CJXU@ntu.edu.sg

Supplementary Methods

Specific assembly protocols of each FNA structures:

- TH21 & TH37: TM buffer (10mM Tris-HCl with 5mM $MgCl_2$ at pH 8) was used as buffer solution to mix all strand components. Sequences can be found in Supplementary Table 3 & 4, respectively. For stability evaluation, sequences for dual-tagged TH21 can be found in Table S11. Mixture was heated to 95°C for 5 min, then rapidly cooled down to 4 °C. Purification was performed through high performance liquid chromatography (HPLC, mobile phase: 25mM Tris, 450mM NaCl, pH 7.2). Assembled TH21 & TH37 were concentrated through Microcon® centrifugal filtration device (30kDa MWCO filters, Amicon).[1, 2]

- TH337: 100μM staple strands were mixed with P8064 scaffold strand in a molar ratio of 10:1 in 0.5x TE buffer (5mM Tris, 1mM EDTA, pH7.9) supplemented with 12mM $MgCl_2$. Sequences can be found in Supplementary Table 5. Assembled TH337 was subsequently purified with Microcon® centrifugal filtration device (100kDa MWCO filters, Amicon) to remove excess staple strands.[3]

- 6H714: Twenty component strands were mixed equally (1μM) in TAE buffer (40mM Tris, 20mM acetic acid, 2mM EDTA, pH 8) containing 125mM magnesium acetate. Sequences can be found in Supplementary Table 6. Mixture was heated to 90°C before slow cooling to 20°C in water bath. Purification was performed through high performance liquid chromatography (HPLC).[4]

- 6H14498 & R13730: short staple strands were mixed with long viral ssDNA M13mp18 as scaffold in 10:1 molar ratio in TAE buffer (40mM Tris, 20mM acetic acid, 2mM EDTA, pH 8) with 12.5 magnesium acetate and annealed from 95°C to 20°C in PCR machine at a rate of 1°C/minute. Sequences can be found in Supplementary Table 7 & 8. Assembled 6H14498 was purified with Microcon® centrifugal filtration device (100kDa MWCO filters, Amicon) to remove excess staple strands.[5, 6]

- B14498 & T14498: short staple strands were mixed with P7560 scaffold strand in 10:1 molar ratio in TE buffer (5mM Tris, 1mM EDTA, pH 8) with 16mM $MgCl_2$. Sequences can be found in Supplementary Table 9 & 10. The mixture was annealed at 85°C for 5min, from 65 to 61°C at -1°C/5min, from 60 to 51°C at -1°C/60min; from 51 to 38°C at -1°C/20min and from 37 to 25°C at -1°C/10min. Assembled B14498 & T14498 was purified by agarose gel and extracted with BioRad Freeze N Squeeze spin columns to remove excess staple strands.[7]

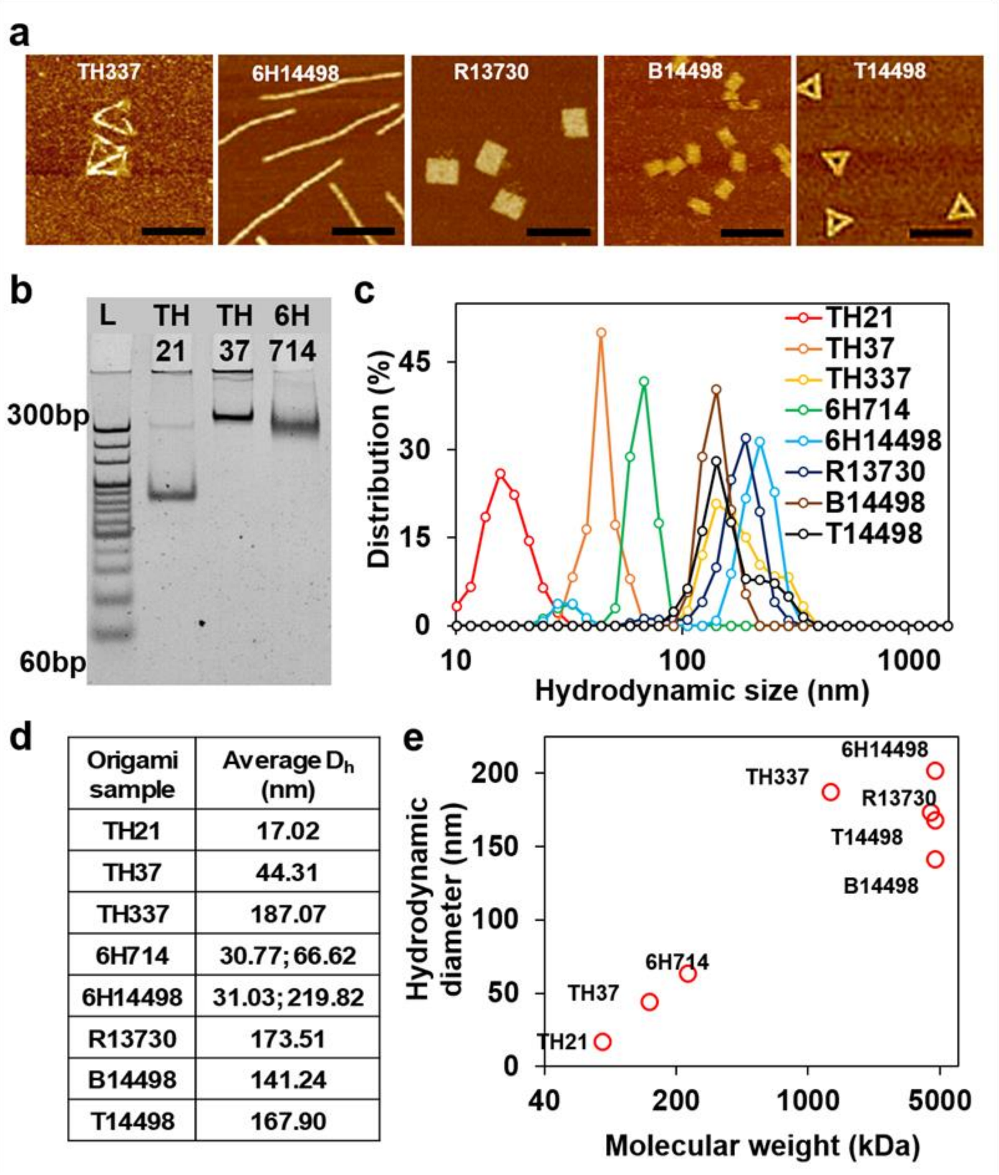

Origami sample	Average D_h (nm)
TH21	17.02
TH37	44.31
TH337	187.07
6H714	30.77; 66.62
6H14498	31.03; 219.82
R13730	173.51
B14498	141.24
T14498	167.90

Supplementary Figure 1. Physical characterization of FNA structures: **a**) AFM visualization of five kinds of FNAs (TH337, 6H14498, R13730, B14498, T14498). **b**) Gel electrophoresis of TH21, TH37 and 6H714. L: 60bp DNA ladder. **c**) Distribution and **d**) average hydrodynamic diameter of synthesized FNAs. 6-helix structures have 2 peaks corresponding to its width and length. **e**) Correlation between average hydrodynamic diameter and molecular weight of distinct FNAs. Source data are provided as a Source Data file. Scale bar: 150nm.

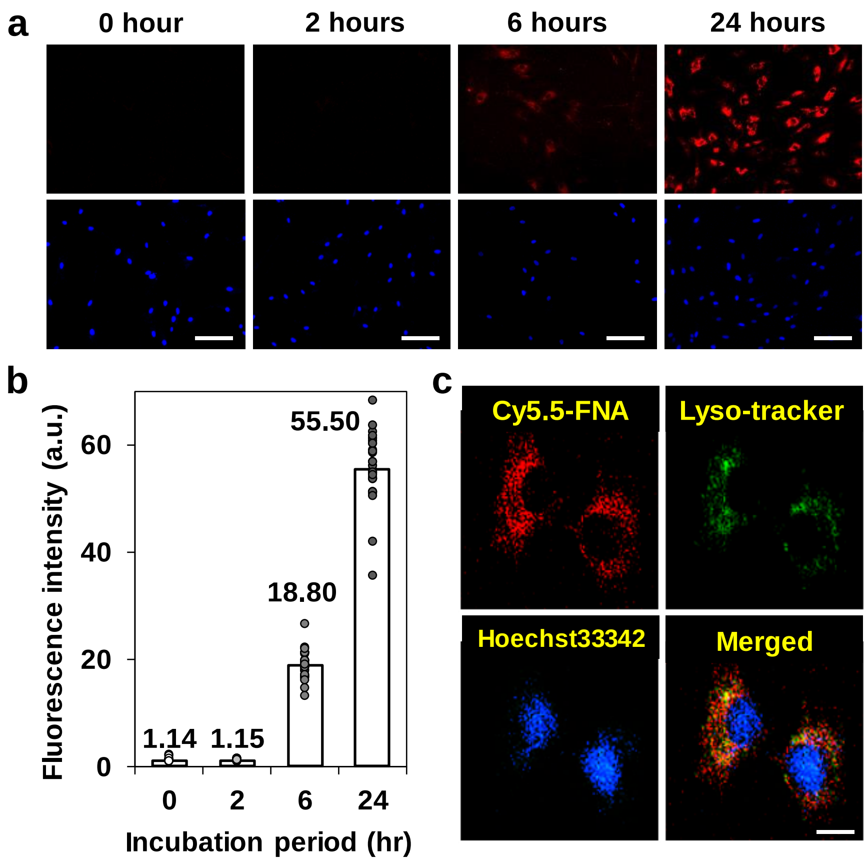

Supplementary Figure 2. Cellular internalization of Cy5.5-tagged TH21 by skin fibroblast cells: **a**) Fluorescence imaging of cell internalization of TH21 after 0, 2, 6 and 24hrs of incubation; scale bar: 50µm. **b**) Quantification of TH21 signal in cells in A (n=30). Each dot on bar charts represents independent sample measurement. **c**) Confocal images of fibroblast cells post the uptake of TH21, nuclear Hoechst33342 staining, and lysosome staining. Non-overlapping Cy5.5 signal shows cytosolic presence of TH21. Source data are provided as a Source Data file. Scale bar: 20µm. Error bars represent standard deviation.

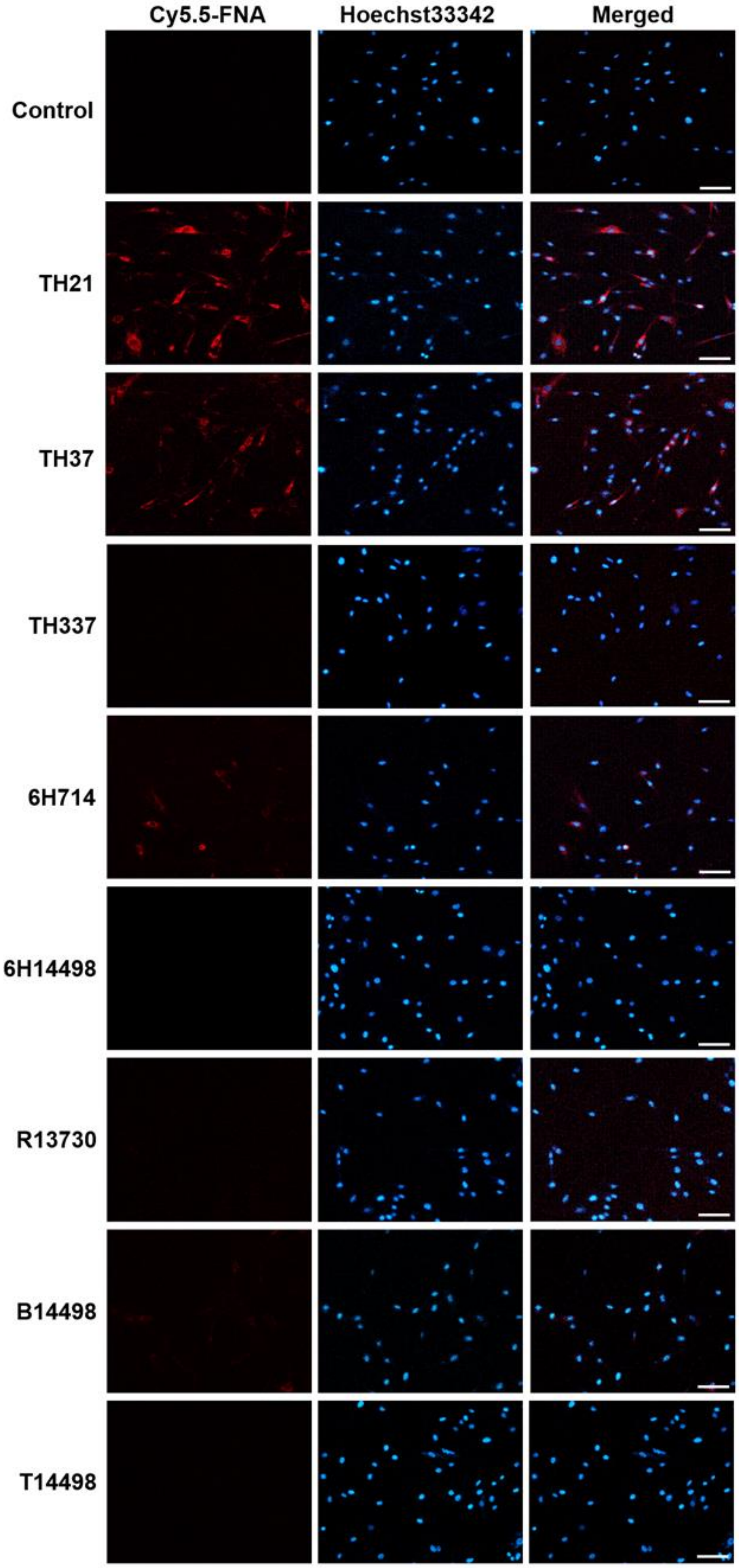

Cy5.5-FNA
Hoechst33342
Merged
Control
TH21
TH37
TH337
6H714
6H14498
R13730
B14498
T14498

Supplementary Figure 3. Representative images of skin fibroblast cells after being incubated with FNAs for 24 hrs. From left to right: Cy5.5-tagged FNAs, Hoechst33342 nuclear staining, and merged channel. Scale bar: 50μm

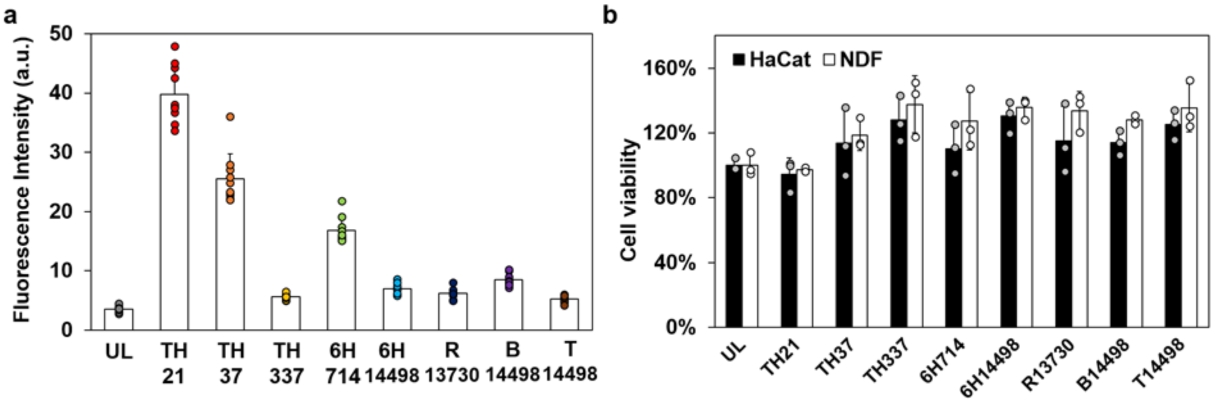

Supplementary Figure 4. *In vitro* evaluation of FNAs on cellular level: **a**) Quantification of cellular FNA fluorescence in Figure S3. Each dot represents value of one sample. **b**) Influence of FNAs (1μg/mL) on the viability of human keratinocytes (HaCat) and human normal dermal fibroblasts (NDF), normalized to unlabeled cells (UL). Each dot on bar charts represents independent sample measurement. Source data are provided as a Source Data file. Error bars represent standard deviation.

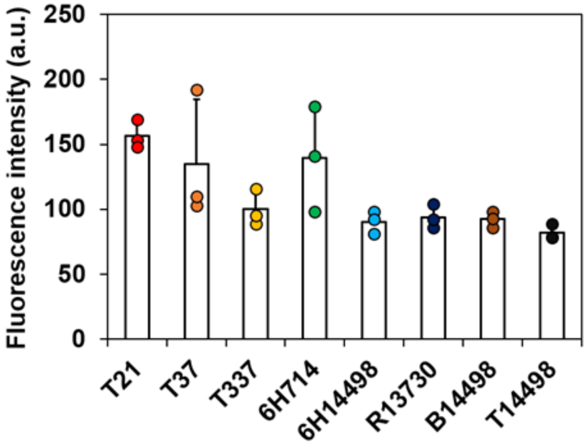

Supplementary Figure 5. Fluorescence intensity of all 8 Cy5.5-FNA nanostructures at the same mass concentration (100 μg/ml). Each dot represents independent sample measurement. Source data are provided as a Source Data file. Error bars represent standard deviation.

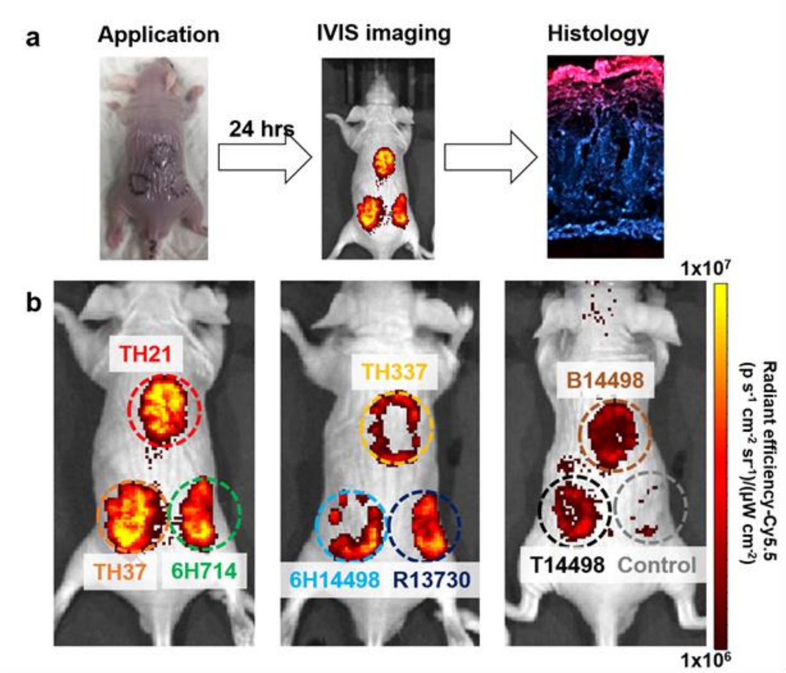

Supplementary Figure 6. *In vivo* FNA penetration study on mice: **a**) Illustration of mice experimental procedure. Following 24hrs of incubation, mice were taken for (*in vivo* imaging) IVIS imaging and sacrificed for subsequent skin histological analysis. **b**) Representative IVIS results of mice post the topical application with Cy5.5-FNAs. Fluorescence at applied site (circled regions) were recorded for analysis.

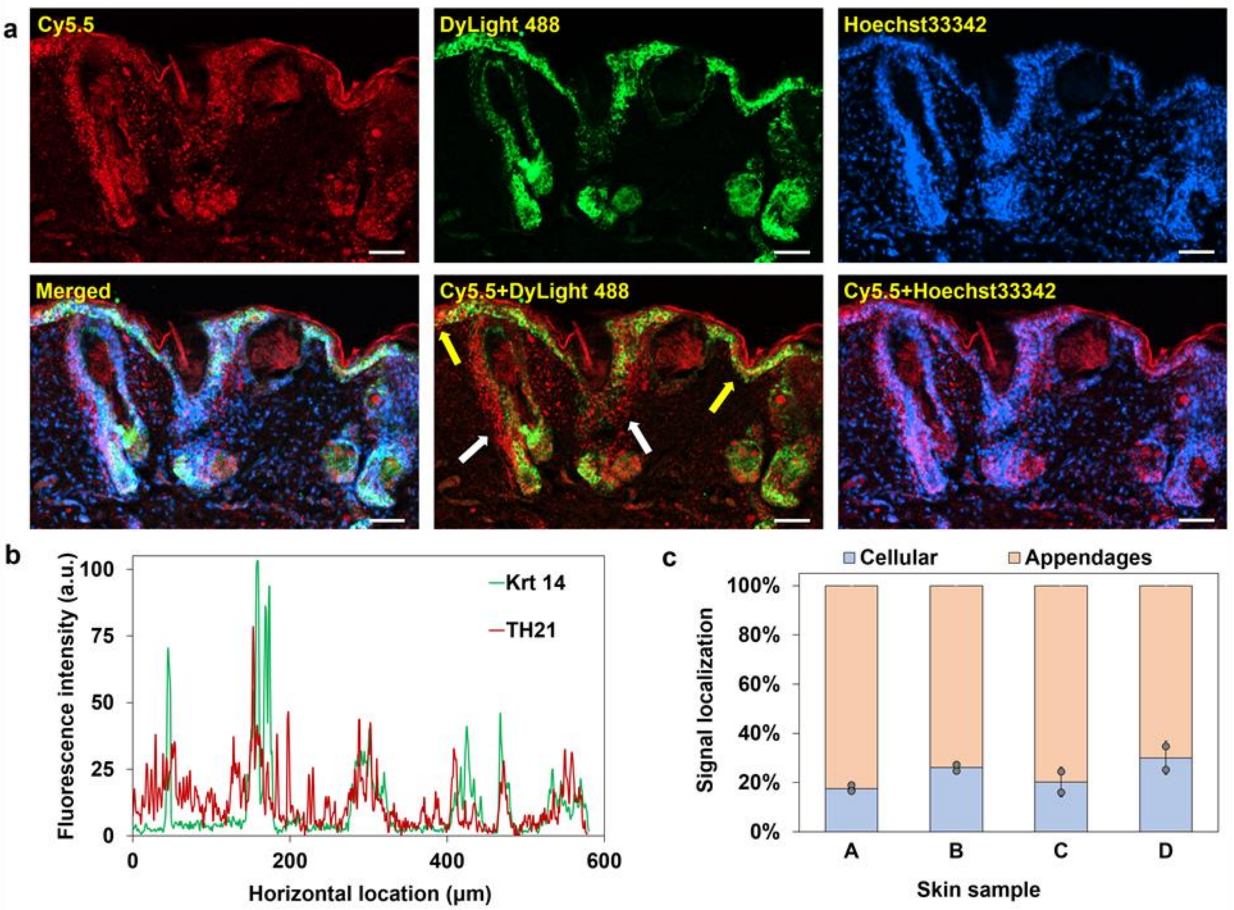

Supplementary Figure 7: Histological analysis of TH21 skin penetration: **a**) Representative fluorescence images of TH21-treated mice skin counter-stained with anti-keratin14. (From left to right) top: Cy5.5-TH21, DyLight488-Krt 14, Hoechst33342 channel; bottom: merged channels, Cy5.5-TH + DyLight488-Krt 14, Cy5.5 + Hoechst3342. White arrow indicates penetration mediated by trans-appendageal route; yellow arrow: through trans/intercellular route. **b**) TH21 and Krt14-stain fluorescence signal profile across same depth. **c**) TH21 localization as determined by correlating both signals in b. Appendages localization is marked with Krt14 expression in a. Each dot represents independent sample measurement. Source data are provided as a Source Data file. Scale bar: 100μm. Error bars represent standard deviation.

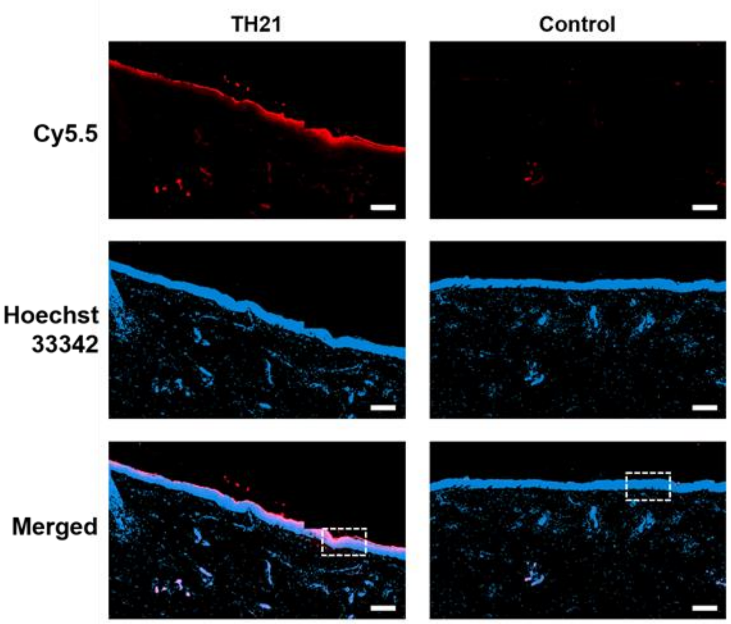

Supplementary Figure 8. Representative fluorescence images showing skin penetration of TH21 in fresh human skin explant: red: Cy5.5-TH21, blue: Hoechst33342 & merged: Cy5.5 + Hoechst3342 channel. Scale bar: 200µm. Dotted regions are viewed closer in Figure 5a.

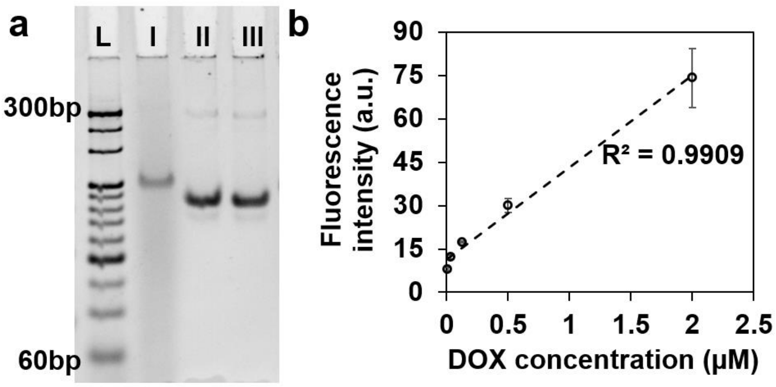

Supplementary Figure 9. Characterization of DOX-loaded TH21 (TH-DOX): **a**) Gel electroporation (left to right: L: 60bp ladder, I: DOX-loaded Cy5.5-tagged TH21, II: Cy5.5-tagged TH21 & III: unmodified TH21); **b**) Fluorescence standard curve for quantification of DOX loading into DNA TH21. Source data are provided as a Source Data file. Error bars represent standard deviation.

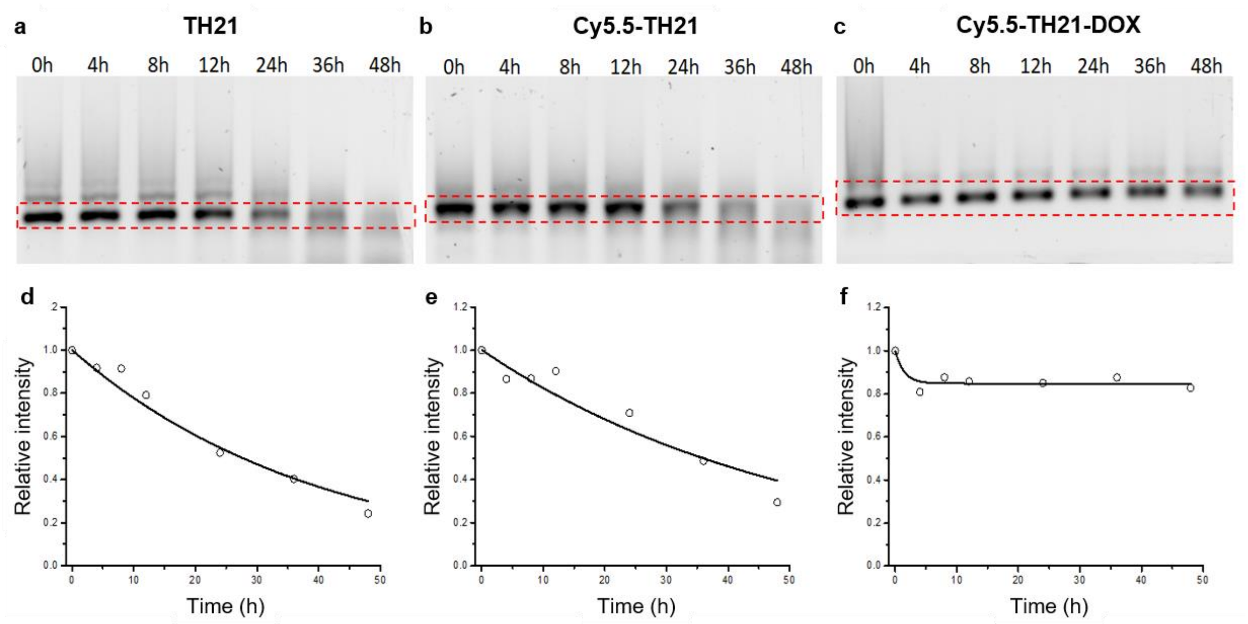

Supplementary Figure 10. Evaluation of TH21 stability in 10% serum condition: Gel electrophoresis study of TH21 (**a**), cy5.5-tagged TH21 (**b**), and cy5.5-tagged DOX-loaded TH21 (**c**) upon different incubation period (0, 4, 8, 12, 24, 36 & 48hrs). (**d**) Quantified band intensity of a showing amount of intact TH21. (**e**) Quantified band intensity of b showing amount of intact cy5.5-TH21. (**f**) Quantified band intensity of c showing amount of intact cy5.5-TH21-DOX. Values are normalized against band intensity at t=0hr.

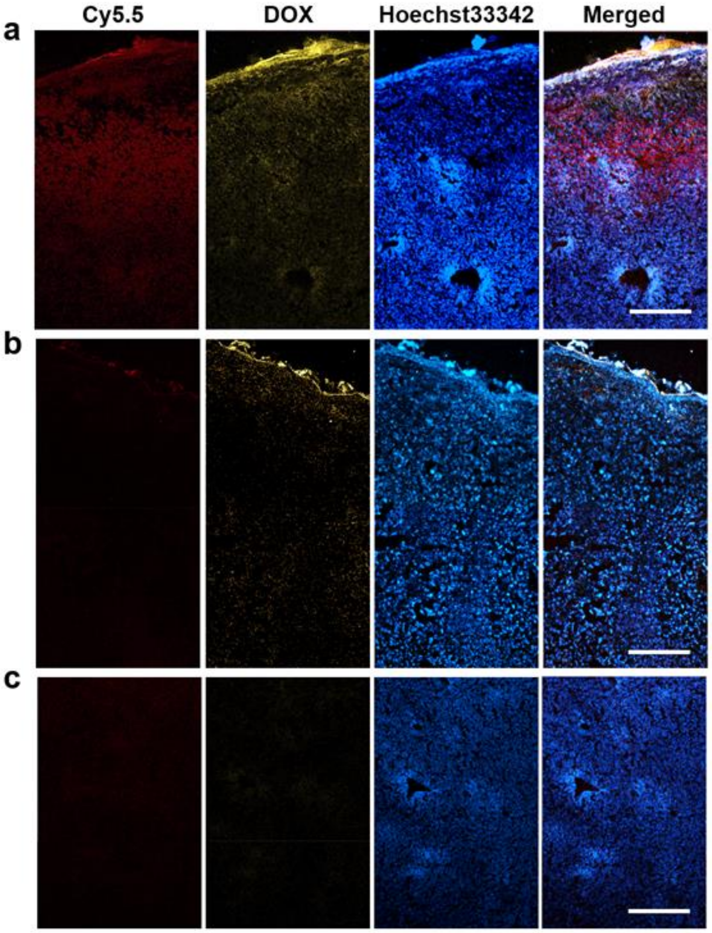

Supplementary Figure 11. Representative fluorescence imaging of skin/tumor histology: **a**) TH-DOX treated, **b**) free DOX-treated, and **c**) control PBS-treated. From left to right: Cy5.5-TH (red channel), DOX (yellow channel), Hoechst33342-stained skin/tumor (blue channel), merged channel. Scale bar: 200μm.

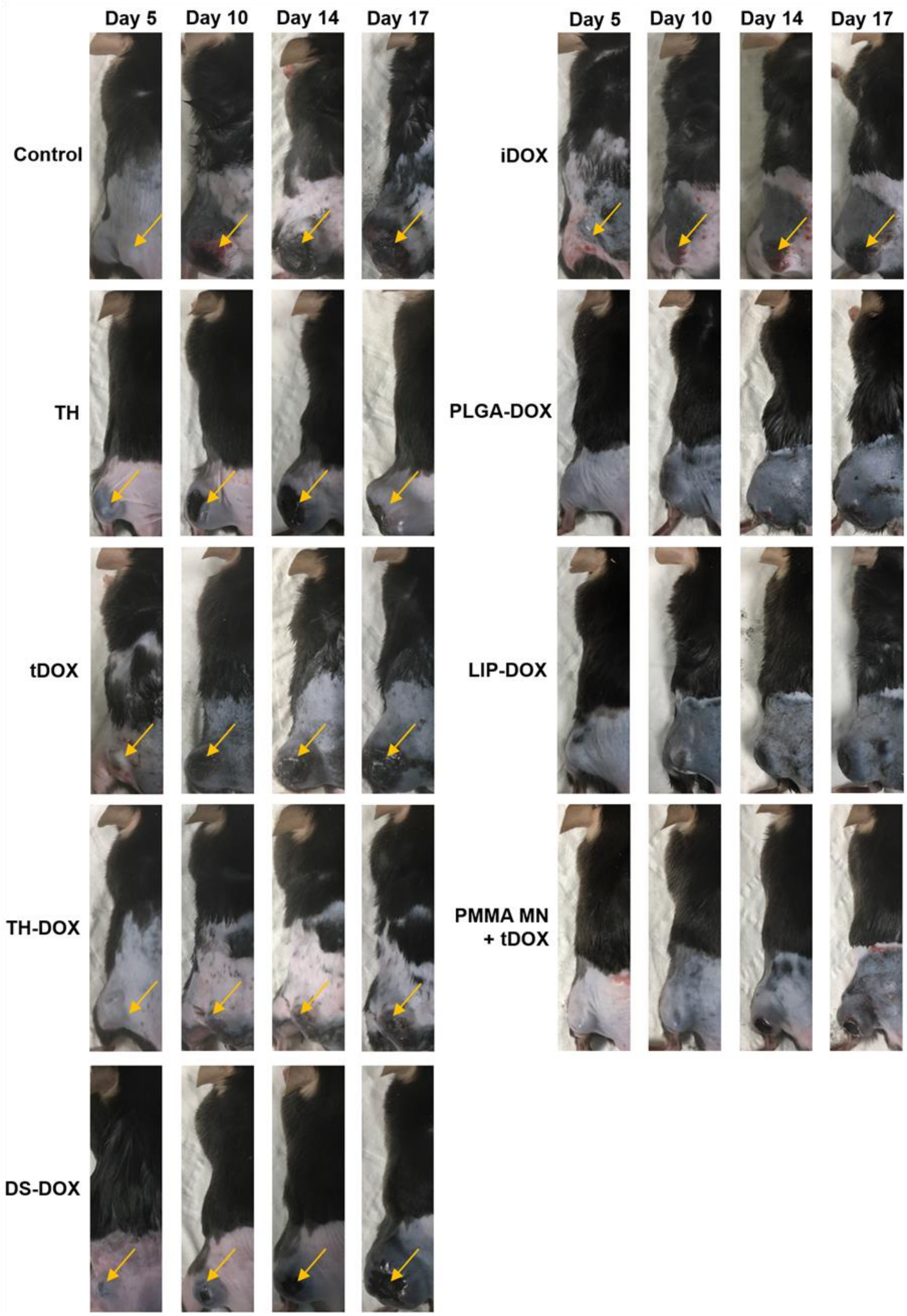
Day 5
Day 10
Day 14
Day 17
Control
TH
tDOX
TH-DOX
DS-DOX
Day 5
Day 10
Day 14
Day 17
iDOX
PLGA-DOX
LIP-DOX
PMMA MN
+ tDOX

Supplementary Figure 12. Representative mice photos showing tumor progression over time (day 5, 10, 14 and 17) following control, TH only, tDOX, TH-DOX, DS-DOX, iDOX, PLGA-DOX, LIP-DOX and PMMA MN + tDOX application. Yellow arrow indicates tumor location.

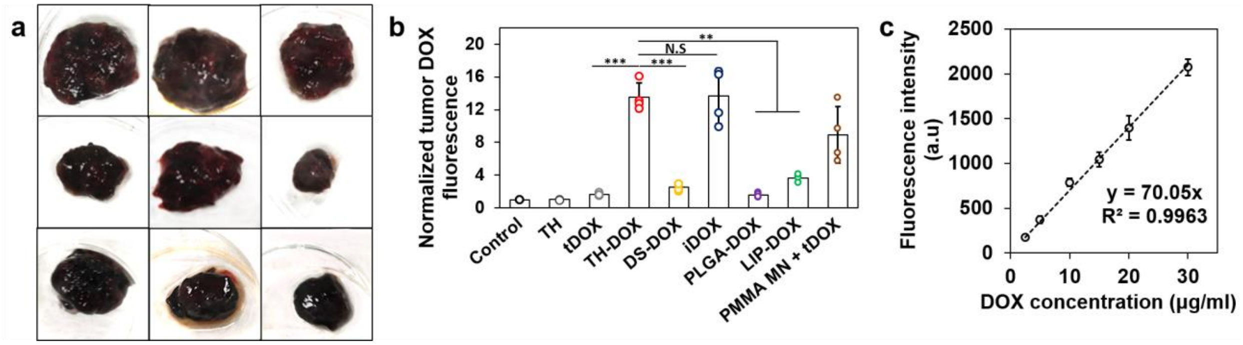

Supplementary Figure 13. Analysis of isolated tumor samples at day 17: **a**) Representative photographical images and **b**) IVIS imaging fluorescence of tumors treated with different DOX formulations (from top left: Control, TH, tDOX, TH-DOX, DS-DOX, iDOX, PLGA-DOX, LIP-DOX & PMMA MN + tDOX). **c**) Standard curve for DOX content following tumor homogenization. Homogenized solution of untreated tumors were spiked with known DOX amount for curve generation. Each dot on bar charts represents independent sample measurement. Source data are provided as a Source Data file. Error bars represent standard deviation. N.S: not significant, **: $p<0.01$, ***: $p<0.001$ (one-way ANOVA).

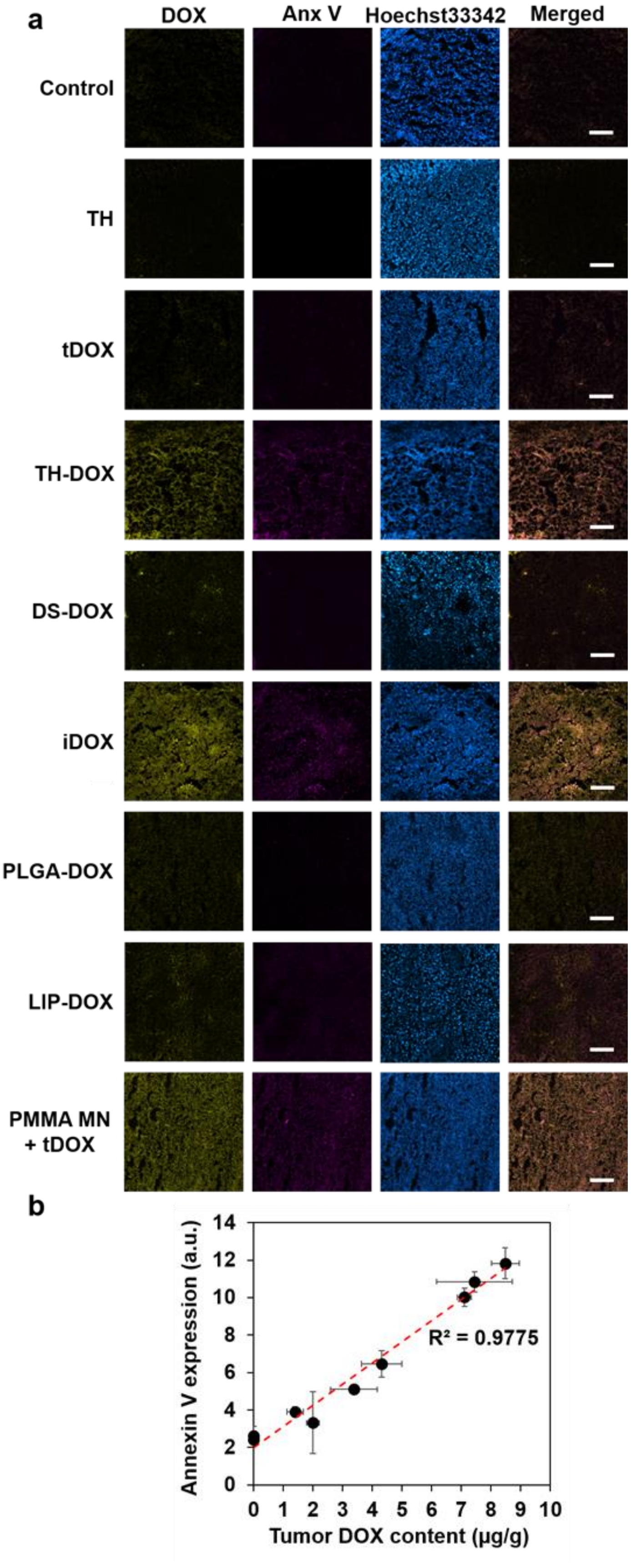
a
DOX
Anx V
Hoechst33342
Merged
Control
TH
tDOX
TH-DOX
DS-DOX
iDOX
PLGA-DOX
LIP-DOX
PMMA MN
+ tDOX
b
Annexin V expression (a.u.)
Tumor DOX content (µg/g)
R² = 0.9775
0
2
4
6
8
10
12
14
0 1 2 3 4 5 6 7 8 9 10

Supplementary Figure 14. Histological examination of isolated tumor samples following distinct DOX treatments: **a**) Representative images showing (from left to right) DOX fluorescence (yellow channel), Annexin V expression (purple channel), corresponding Hoechst33342 nuclear fluorescence (blue channel) and merged DOX & Annexin V channel. **b**) Correlation between DOX content and Annexin V expression in treated tumor samples. Scale bar: 100µm. Source data are provided as a Source Data file. Error bars represent standard deviation.

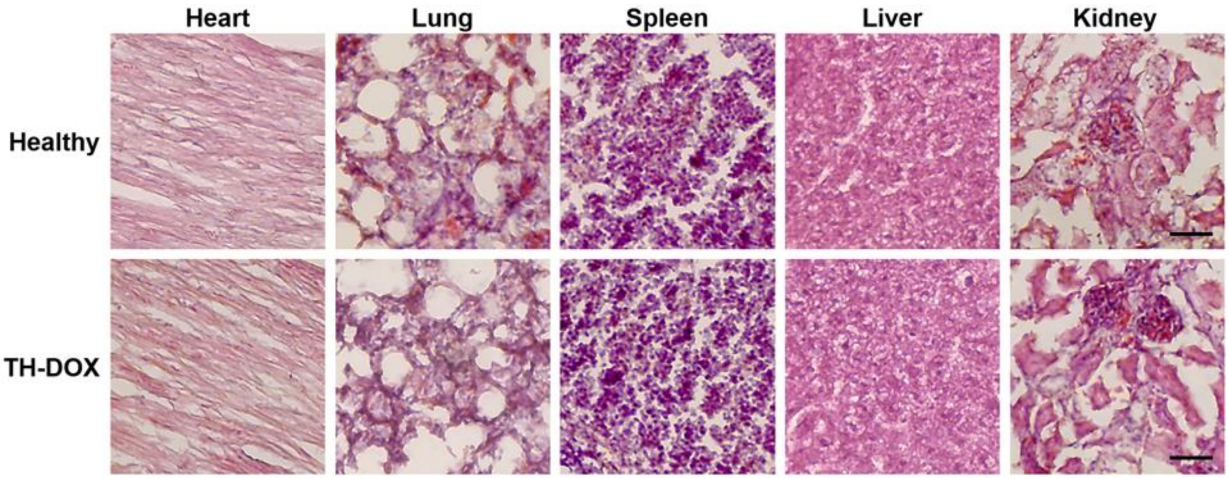

Supplementary Figure 15. Hematoxylin and Eosin staining of the major organs from healthy or TH-DOX treated mice. Scale bar: 50µm.

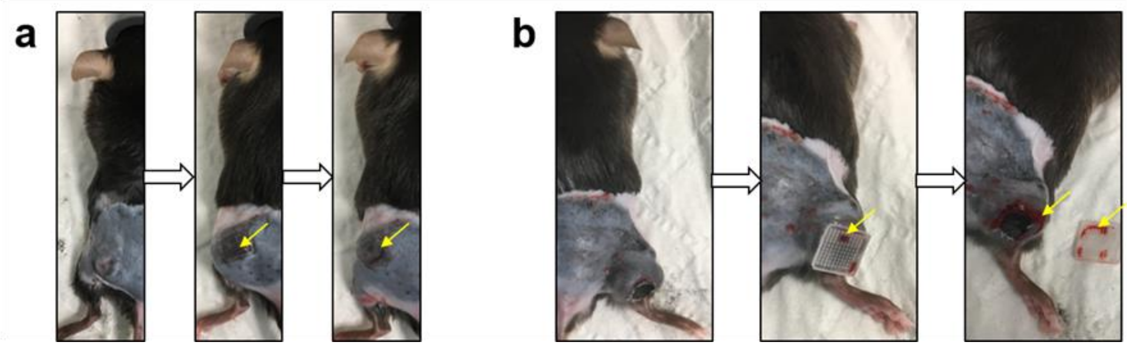

Supplementary Figure 16. Application of MN on mice tumor: **a**) routine and **b**) injury-causing MN application at early or later-stage tumor development. From left to right: tumor prior MN application, 1min application, tumor after MN application. Bleeding was noted on later-stage tumor application (yellow arrow).

Supplementary Table 1. FNA nanostructures in this study

No.	DNA origami structure	Abbre-viation	Schematic	Molecular Weight	Buffer Conditions
1	Tetrahedron 21bp	TH21	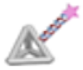	81.9 kDa	pH 8.0, 10mM Tris, 5mM $MgCl_2$
2	Tetrahedron 37bp	TH37	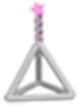	144.3 kDa	pH 8.0, 10mM Tris, 5mM $MgCl_2$
3	Tetrahedron 337bp	TH337	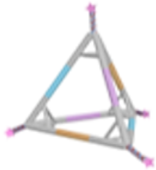	1,314.3 kDa	pH 8.0, 5mM Tris, 1mM EDTA, 12mM $MgCl_2$
4	6-Helix 714bp	6H714	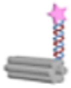	232 kDa	pH 8.0,40mM Tris, 2mM EDTA, 125mM $(CH_3COO)_2Mg$
5	6-Helix 14498bp	6H14498	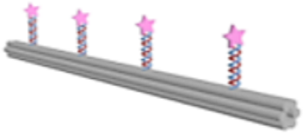	4,711.9 kDa	pH 8.0,4mM Tris, 2mM EDTA, 12.5mM $(CH_3COO)_2Mg$
6	Rectangle plane 13730bp	R13730	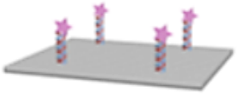	4,462.3 kDa	pH 8.0,4mM Tris, 2mM EDTA, 12.5mM $(CH_3COO)_2Mg$
7	Box 14498bp	B14498	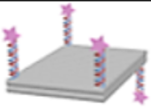	4,711.9 kDa	pH=8.0, 5mM Tris,1mM EDTA, 16mM $MgCl_2$
8	Triangle plane 14498bp	T14498	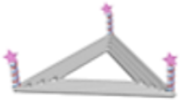	4,711.9 kDa	pH=8.0, 40mM Tris, 20mM Acetic Acid, 2mM EDTA, 12.5 mM $(CH_3COO)_2Mg$

Supplementary Table 2. Sequences for cy5.5-tagged TH21

TH-S1	TTTTTTTTTTTTTTTTTTTTCAGTTGAGACGAACATTCCTAAGTCTGAAATTTATC ACCCGCCATAGTAGACGTATCACCAGG
TH-S2	GCTACACGATTCAGACTTAGGAATGTTCGACATGCGAGGGTCCAATACCGACGA TTACAGCTT
TH-S3	GTGATAAAACGTGTAGCAAGCTGTAATCGACGGGAAGAGCATGCCCATCCACT ACTATGGCGG
TH-S4	CTCGCATGACTCAACTGCCTGGTGATACGAGGATGGGCATGCTCTTCCCGACGG TATTGGACC
Cy5.5	AAAAAAAAAAAAAAAAAAAA

Supplementary Table 3. Sequences for cy5.5-tagged TH37

A37.1	TTTTTTTTTTTTTTTTTTTTCCCTGTACTGGCTAGGAATTCACGTTTTAATCTGGGC TTT GGGTTAAGAAACTCCCCG
A37.2	CGCTGGAGGCGCATCACCG TTT GCGTATGTGTTCTGTGCGGCCTGCCGTCCCGTGTGGG
B37.1	CGGTGATGCGCCTCCAGCGCGGGGAGTTTCTTAACCC TTT CCGACTTACAAGAGCCGG
B37.2	GCGAGACTCAGGTGGTGCC TTT GGCATTCGACCAGGAGATATCGCGTTCAGCTATGCCC
C37.1	CCCATGAGAATAATACCGCCGATTTACGTCAGTCCGG TTT CCCACACGGGACGGCAGGC
C37.2	CGCACAGAACACATACGC TTT GGGCATAGCTGAACGCGATATCTCCTGGTCGAATGCC
D37.1	GCCCAGATTAAAACGTGAATTCCTAGCCAGTACAGGG TTT CCGGACTGACGTAAATCGG
D37.2	CGGTATTATTCTCATGGG TTT GGCACCACCTGAGTCTCGCCCGGCTCTTGTAAGTCGG
Cy5.5	AAAAAAAAAAAAAAAAAAAA

Supplementary Table 4. Sequences for cy5.5-tagged TH337

Scaffold	P8064 scaffold strand
tetrahedron-8[44]	TGTCAGGGTGGCGGTCCACGCTGGATCCTTTTTTTTTTTTTTTTTTT
Cy5.5	AAAAAAAAAAAAAAAAAAA

Supplementary Table 5. Sequences for cy5.5-tagged 6H714

six-helix-1	CAGTTGACTGCTAGTACCTGAGCACTGAATGCGATGTAGAAGTAGCTCTGCTCCATC
six-helix-2	CGACTTGATGGAGCAGACCTATCGTCAC
six-helix-3	AGGCAGATACGAAGAGCGAGGCTATGTCGTAGATAGTTCTCGCACGACGCTAGACAC
six-helix-4	GCAGTCAACTGAAAAAAAAAAAAAAAAAAAA
six-helix-5	CGGTACGTGACGATAGGACACATCAGATGTCTTAGGAGAGGTCACAGTAACCTTCGACAATCT
six-helix-6	AGATTGTCGAACGTATCTGCCT
six-helix-7	GATGCGTGAACTATGACATCTGATGTGTGCTACTTGTCACCA
six-helix-8	TCGAGTACGCTCTTGGTTACTGTGACCTGTGCTCACGAGGAC
six-helix-9	GCATTCACTCCTAACTACGAC
six-helix-10	TCTAGCGTGTCTAGCGT
six-helix-11	TGTCCGTGCAACCGATCAATCC
six-helix-12	GCCTAGCGATCCAATGGAACGACCGTATTGCTGAGGTGAGTGTATGTATCACTTGCACGGACA
six-helix-13	CTGTACCGTTG
six-helix-14	GGATTGATCGGATGCCAGACGCATCGGATTCGATGAGCCTACTCGACCAACTCAACG
six-helix-15	GGCAATGTCCACCATTGGATCG

six-helix-16	CAACGGTACAGTGGTGACTCCAACCTTGTAACGTCCTCGATAACGCTGGACATTGCC
six-helix-17	CGTGCGACTGGCATGTGATACATACACTGGTTGGACTACATC
six-helix-18	ATAGCCTGGCTCATGCAATACGGTCGTTGCGTTATGGTACTA
six-helix-19	GTTACAACACCTCACGAATCC
six-helix-20	GCACCACGTTGAGTTGG
Cy5.5	TTTTTTTTTTTTTTTTTTTT

Supplementary Table 6. Sequences for cy5.5-tagged 6H14498

Scaffold	viral ssDNA M13mp18
bundle-1[7]-3[14]	TCAAAATGCCCGTACCCCTGCTTTTTTTTTTTTTTTTTTTT
bundle-4[321]-0[308]	CATTAACCAACGCCCCGGAACCAGAGCCCCTTGCTTGAAAGGTTTTTTTTTTTTTTTTTTTT
bundle-4[615]-0[602]	GGTCAGGACAATGACATTCAACCGATTGTAAGGCGAACAGAGTTTTTTTTTTTTTTTTTTTT
bundle-4[1161]-0[1148]	ATTGTGAAATAAGGGTCTTTCCAGAGCCAGGTTTTCGGGGAATTTTTTTTTTTTTTTTTTTT
Cy5.5	AAAAAAAAAAAAAAAAAAAA

Supplementary Table 7. Sequences for cy5.5-tagged R13730

Scaffold	viral ssDNA M13mp18
rectangle-79	TGAGGCAGGCGTCAGACTGTAGCGTAGCAAGGTTTTTTTTTTTTTTTTTTTT
rectangle-95	CGGAATTATTGAAAGGAATTGAGGTGAAAAATTTTTTTTTTTTTTTTTTTT
rectangle-200	GCTCCATGAGAGGCTTTGAGGACTAGGGAGTTTTTTTTTTTTTTTTTTTT
rectangle-184	GCCAGCTGCCTGCAGGTCGACTCTGCAAGGCGTTTTTTTTTTTTTTTTTTTT
Cy5.5	AAAAAAAAAAAAAAAAAAAA

Supplementary Table 8. Sequences for cy5.5-tagged B14498

Scaffold	P7560 scaffold strand
0-0	AGCCTGGGCCTGGCCCTGAGATCTATCAGGCGATGATAAATCTTTGTCGCTCTCTCAAGTAGAAT
0-189	TGAGTTCGGTCATAGCCCGCCATCTTTTCATATTTGTCGCTCTCTCAAGTAGAAT
20-0	GCTTCAACCAGACCTTCTGCGAACGAGTTTTGTCGCTCTCTCAAGTAGAAT
19-192	ATAACCTTGCTTCTGTAAATCATGTAAATGCTTTTGTCGCTCTCTCAAGTAGAAT
Cy5.5	TTTTATTCTACTTGAGAGAGCGAC

Supplementary Table 9. Sequences for cy5.5-tagged T14498

Scaffold	P7560 scaffold strand
A65	TGCTATTTTGCACCCAGCTACAATTTTGTTTTGAAGCCTTAAATTTGTCGCTCTCTC AAGTAGAAT
B65	CCTGACGAGAAACACCAGAACGAGTAGGCTGCTCATTCAGTGATTTGTCGCTCTCT CAAGTAGAAT
C65	ACGTGGACTCCAACGTCAAAGGGCGAATTTGGAACAAGAGTCCTTTGTCGCTCTCT CAAGTAGAAT
Cy5.5	TTTTATTCTACTTGAGAGAGCGAC

Supplementary Table 10. Sequences for dual cy3, cy5-tagged TH and TH'

Cy3-TH-S1	[Cyanine3]-ATTGCTGTATTGGCTCTGGTGATGCGTTAAAGGATCTCGTATAGCAGCTCAGTC CACTCGAAC
Cy5-TH-S2	[Cyanine5]-CATAGTCAATAACGCATCACCAGAGCCAAATGACGACATCTGTGCGATGAAAC CTAGCAGACC
Cy5-TH-S2-BHQ3	[Cyanine5]-CATAGTCAATAACGCATCACCAGAGCCAAATGACGACATCTGTGCGATGAAAC CTAGCAGACC-[BHQ3]
TH-S3	GAGATCCTATGACTATGGGTCTGCTAGGTACAGTCTGTCGCTTATGCACTAGAG CTGCTATAC
TH-S4	TGTCGTCAAACAGCAATGTTCGAGTGGACAAGTGCATAAGCGACAGACTGATC ATCGCACAGA

Supplementary References

1. Lin, M. et al. Electrochemical detection of nucleic acids, proteins, small molecules and cells using a DNA-nanostructure-based universal biosensing platform. *Nat. Protoc.* **11**, 1244 (2016).
2. Xing, S. et al. Constructing higher-order DNA nanoarchitectures with highly purified DNA nanocages. *ACS Appl. Mater. Interfaces* **7**, 13174-13179 (2014).
3. Iinuma, R. et al. Polyhedra self-assembled from DNA tripods and characterized with 3D DNA-PAINT. *Science*, 1250944 (2014).
4. Liu, P. et al. Charge Neutralization Drives the Shape Reconfiguration of DNA Nanotubes. *Angew. Chem. Int. Ed.* **130**, 5516-5520 (2018).
5. Stearns, L.A. et al. Template-directed nucleation and growth of inorganic nanoparticles on DNA scaffolds. *Angew. Chem. Int. Ed.* **121**, 8646-8648 (2009).
6. Rothemund, P.W. Folding DNA to create nanoscale shapes and patterns. *Nature* **440**, 297 (2006).
7. Zhou, C., Duan, X. & Liu, N. A plasmonic nanorod that walks on DNA origami. *Nat. Commun.* **6**, 8102 (2015).
