# Supplementary material for: Framework nucleic acids as programmable carrier for transdermal drug delivery
Source: Nat Commun. 2019 Mar 8;10:1147. doi: 10.1038/s41467-019-09029-9 (PMC6408537; doi:10.1038/s41467-019-09029-9)
Supplement: Supplementary file 3 — Reporting Summary [file 41467_2019_9029_MOESM3_ESM.pdf]

nature research

Corresponding author(s): Chenjie XU, Chunhai FAN

Reporting Summary

Nature Research wishes to improve the reproducibility of the work that we publish. This form provides structure for consistency and transparency in reporting. For further information on Nature Research policies, see Authors & Referees and the Editorial Policy Checklist.

Statistical parameters

When statistical analyses are reported, confirm that the following items are present in the relevant location (e.g. figure legend, table legend, main text, or Methods section).

n/a	Confirmed	
☐	☒	The exact sample size (*n*) for each experimental group/condition, given as a discrete number and unit of measurement
☐	☒	An indication of whether measurements were taken from distinct samples or whether the same sample was measured repeatedly
☐	☒	The statistical test(s) used AND whether they are one- or two-sided<br>*Only common tests should be described solely by name; describe more complex techniques in the Methods section.*
☐	☒	A description of all covariates tested
☐	☒	A description of any assumptions or corrections, such as tests of normality and adjustment for multiple comparisons
☐	☒	A full description of the statistics including central tendency (e.g. means) or other basic estimates (e.g. regression coefficient) AND variation (e.g. standard deviation) or associated estimates of uncertainty (e.g. confidence intervals)
☐	☒	For null hypothesis testing, the test statistic (e.g. *F*, *t*, *r*) with confidence intervals, effect sizes, degrees of freedom and *P* value noted<br>*Give P values as exact values whenever suitable.*
☒	☐	For Bayesian analysis, information on the choice of priors and Markov chain Monte Carlo settings
☒	☐	For hierarchical and complex designs, identification of the appropriate level for tests and full reporting of outcomes
☐	☒	Estimates of effect sizes (e.g. Cohen's *d*, Pearson's *r*), indicating how they were calculated
☐	☒	Clearly defined error bars<br>*State explicitly what error bars represent (e.g. SD, SE, CI)*

Our web collection on statistics for biologists may be useful.

Software and code

Policy information about availability of computer code

Data collection	Living Image software (in vivo imaging systems); Zeiss Zen blue (confocal imaging); Gen5 (microplate reader)
Data analysis	Living Image software; Zeiss Zen blue; ImageJ; IBM SPSS statistics 23; Microsoft excel

For manuscripts utilizing custom algorithms or software that are central to the research but not yet described in published literature, software must be made available to editors/reviewers upon request. We strongly encourage code deposition in a community repository (e.g. GitHub). See the Nature Research guidelines for submitting code & software for further information.

Data

Policy information about availability of data

All manuscripts must include a data availability statement. This statement should provide the following information, where applicable:
- Accession codes, unique identifiers, or web links for publicly available datasets
- A list of figures that have associated raw data
- A description of any restrictions on data availability

The authors declare that all data supporting the findings of this study are available within the paper and its supplementary information files

Field-specific reporting

Please select the best fit for your research. If you are not sure, read the appropriate sections before making your selection.

☒ Life sciences ☐ Behavioural & social sciences ☐ Ecological, evolutionary & environmental sciences

For a reference copy of the document with all sections, see nature.com/authors/policies/ReportingSummary-flat.pdf

Life sciences study design

All studies must disclose on these points even when the disclosure is negative.

Sample size	All in vitro analyses involve at least 8 independent samples and in vivo analyses involve at least 4 samples with no outliers. Such sample size was chosen with referral to similar studies previously
Data exclusions	No data were excluded from the analyses, except for outliers as determined with Tukey's fences
Replication	All in vitro analyses were repeated at least three times with n>=3; all in vivo analyses were repeated at least twice
Randomization	Animals, ex vivo tissues were allocated randomly to different treatment groups
Blinding	Single blinding was performed for all animal studies, where personnel in charge of data collection and analyses is not aware of treatment conditions

Reporting for specific materials, systems and methods

Materials & experimental systems

n/a	Involved in the study	
☐	☒	Unique biological materials
☐	☒	Antibodies
☒	☐	Eukaryotic cell lines
☒	☐	Palaeontology
☐	☒	Animals and other organisms
☒	☐	Human research participants

Methods

n/a	Involved in the study	
☒	☐	ChIP-seq
☒	☐	Flow cytometry
☒	☐	MRI-based neuroimaging

Unique biological materials

Policy information about availability of materials

Obtaining unique materials	All unique materials used are available from the authors or from standard commercial sources (Sigma-Aldrich, Lonza, Gibco Thermofisher)

Antibodies

Antibodies used	mouse Cytokeratin14 antibody (KRT14/532) [DyLight 488] from Novus Biologicals
Validation	Novusbio guarantees primary and secondary antibodies of homology >90% within one year from receipt. Authors validated and optimized Antibody concentration with signal on dermis region

Animals and other organisms

Policy information about studies involving animals; ARRIVE guidelines recommended for reporting animal research

Laboratory animals	Males, 6 week-old NCR nude mice or C57Bl6 mice were obtained from InVivos Singapore, used within 2 weeks of delivery
Wild animals	No wild animals were used in this study
Field-collected samples	This study does not involve field collection of samples
